# Bioethanol Production From H_2_/CO_2_ by Solventogenesis Using Anaerobic Granular Sludge: Effect of Process Parameters

**DOI:** 10.3389/fmicb.2021.647370

**Published:** 2021-03-10

**Authors:** Yaxue He, Chiara Cassarini, Piet N. L. Lens

**Affiliations:** National University of Ireland Galway, Galway, Ireland

**Keywords:** ethanol, H_2_/CO_2_, bioconversion, temperature, trace metals

## Abstract

CO_2_ fermentation by biocatalysis is a promising route for the sustainable production of valuable chemicals and fuels, such as acetic acid and ethanol. Considering the important role of environmental parameters on fermentation processes, granular sludge from an industrial anaerobic wastewater treatment system was tested as inoculum for ethanol production from H_2_/CO_2_ at psychrophilic (18°C), submesophilic (25°C), and mesophilic (30°C) temperatures. The highest acetic acid and ethanol production was obtained at 25°C with a final concentration of 29.7 and 8.8 mM, respectively. The presence of bicarbonate enhanced acetic acid production 3.0 ∼ 4.1-fold, while inhibiting ethanol production. The addition of 0.3 g/L glucose induced butyric acid production (3.7 mM), while 5.7 mM ethanol was produced at the end of the incubation at pH 4 with glucose. The addition of 10 μM W enhanced ethanol production up to 3.8 and 7.0-fold compared to, respectively, 2 μM W addition and the control. The addition of 2 μM Mo enhanced ethanol production up to 8.1- and 5.4-fold compared to, respectively, 10 μM Mo and the control. This study showed that ethanol production from H_2_/CO_2_ conversion using granular sludge as the inoculum can be optimized by selecting the operational temperature and by trace metal addition.

## Graphical Abstract

Bioethanol from H2/CO2 by solventogenesis using anaerobic granular sludges.

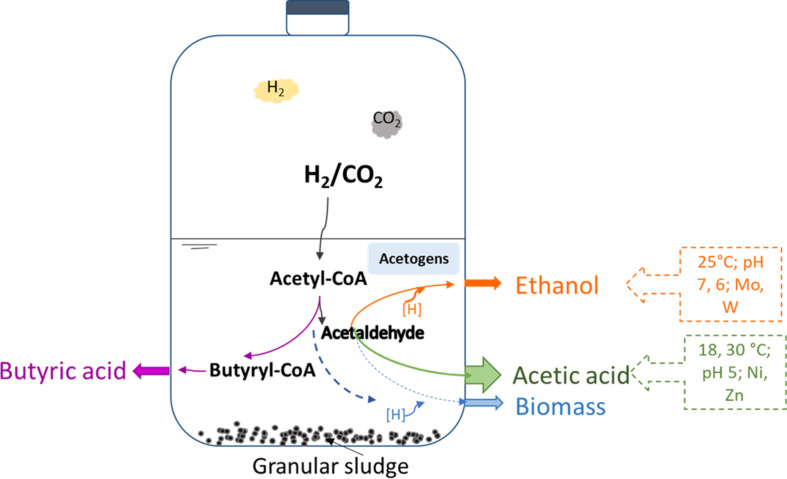

## Highlights

- Submesophilic temperatures (25°C) and an initial pH 6 enhanced ethanol production.
- Increased ratio of CO_2_/H_2_ by bicarbonate addition enhanced acetic acid production (3.0 ∼ 4.1-fold), but inhibited ethanol production.
- Glucose addition (0.3 g/L) enhanced butyric acid production (3.3 mM), while ethanol production occurred at pH 4.
- Addition of 10 μM W or 2 μM Mo enhanced ethanol production up to 7.0 or 5.4-fold, respectively.

## Introduction

The increasing demand for fuel energy and its gradual depletion renders the development of renewable energy necessary and emergent (Devarapalli and Atiyeh, 2015). An innovative solution is to use C_1_ gasses (i.e., one carbon atom gasses) as the substrate to produce valuable chemicals, e.g., volatile fatty acids (VFAs) as well as ethanol and butanol by microbes (Sadhukhan et al., 2016; Fernández-Naveira et al., 2017a). These C_1_ compounds include carbon monoxide (CO), carbon dioxide (CO_2_), methane (CH_4_), and synthesis gas (CO/CO_2_ and H_2_ mixtures) produced from biomass and domestic/agricultural wastes. Besides, H_2_ becomes available from the conversion of excess power produced by renewable energy sources, such as wind and solar power, which face challenges of balancing power production and demand. The generation of valuable chemicals and fuels from H_2_/CO_2_ and syngas (mainly containing CO, H_2_ and CO_2_) fermentation is economic and has sustainable advantages (Burk et al., 2014) compared to traditional corn (Eisentraut, 2010; Mohammadi et al., 2011) or cellulosic material (Naik et al., 2010) fermentation. Moreover, ethanol has a higher energy density and easier storage and transportability than H_2_ (Pereira, 2013; Sarkar et al., 2017). Homoacetogenesis and solventogenesis from H_2_/CO_2_ occur according to reactions 1 and 2:

(1) $$2\,C\,O_{2}\,\left(g\right)+4\,H_{2}\,\left(g\right)\to C\,H_{3}\,C\,O\,O\,H\,\left(l\right)+2\,H_{2}\,O\,\left(l\right)△\,G_{r}^{\theta }-75.4\,\mathrm{kJ}/\mathrm{moL}$$

(2) $$2\,C\,O_{2}\,\left(g\right)+6\,H_{2}\,\left(g\right)\to C\,H_{3}\,C\,H_{2}\,O\,H\,\left(l\right)+3\,H_{2}\,O\,\left(l\right)△\,G_{r}^{\theta }-96.5\,\mathrm{kJ}/\mathrm{moL}$$

with $△\,{\text{G}}_{\text{r}}^{\theta }$ is the standard reaction Gibbs energy, T = 298.15K and P = 100kPa.

Granular sludge from Upflow Anaerobic Sludge Bed (UASB) wastewater treatment plants can be used as inoculum for VFAs (Dogan et al., 2005) and ethanol (Steinbusch et al., 2008) production. UASB sludge consists of mixed microbial communities and its full-scale applications have less contamination problems compared to pure cultures. Temperature is an important factor influencing fermentation, for example, mesophilic conditions are optimum for homoacetogen *Clostridium* sp. in syngas fermentation (Singla et al., 2014; Shen et al., 2017). Limited studies focus on psychrophilic or submesophilic conditions for alcohol production from C_1_ gas or syngas by mixed cultures (Liu et al., 2018). Instead, substantial studies focused on mesophilic conditions despite submesophilic conditions are with merits of low energy consumption for high temperature control (Ramió-Pujol et al., 2015). Also the pH can significantly affect both biomass growth and the product formation rate. As the external pH begins to drop due to acid accumulation, an organism may begin to produce alcohols to prevent a further drop in pH (Padan et al., 1981; Cotter et al., 2009). Glucose addition to the medium can enhance alcohol production via overexpressing the ferredoxin-dependent aldehyde oxidoreductase (AOR) gene in *Clostridium carboxidivorans* (Cheng et al., 2019). AOR is involved in conversion of carboxylic acids into their corresponding alcohols without ATP consumption in acetogens such as *C. ljungdahlii* and *C. autoethanogenum* (Liu et al., 2012; Cheng et al., 2019). Besides, glucose offers extra carbon source and releases CO_2_ during the glycolysis pathway, which can be re-assimilated via the Wood Ljungdahl pathway (WLP) with H_2_ as electron donor and thus enhance the carbon conversion efficiency.

Acetic acid and ethanol are produced from CO_2_ by acetogens via the Wood-Ljungdahl pathway (WLP) catalyzed by different enzymes (Fast and Papoutsakis, 2012). Formate dehydrogenase (FDH) is one of the key enzymes in the WLP, converting CO_2_ into acetyl-CoA and leading to the production of acetate. Acetate yields acetaldehyde catalyzed by ferredoxin aldehydeoxydoreductase (AOR). Then, ethanol is produced through the reduction of acetaldehyde by alcohol dehydrogenase (ADH) catalyzing the reduction of acetyl CoA to ethanol (Andreesen and Makdessi, 2008; Jiann-Shin, 2010). Several studies compared the effect of trace metal addition on ethanol production from syngas in pure cultures of *Clostridium ragsdalei* (Saxena and Tanner, 2011). The presence of W enhances ethanol production compared to the absence of W from carbon monoxide by anaerobic granular sludge (Chakraborty et al., 2020). Molybdate (Mo) is an analog of tungsten (W) and binds in the active sites of some enzymes, such as AOR and ADH (Fernández-Naveira et al., 2017b). Other trace metals like zinc (Zn) and nickel (Ni) can stimulate alcohol production by enhancing the FDH and ADH synthesis and activity (Yamamoto et al., 1983).

Based on our previous study on the optimization of ethanol production from H_2_/CO_2_, the highest ethanol production was reached at 25°C compared to 37 and 55°C by anaerobic granular sludge (He et al., 2020). This study further investigated homoacetogenesis and solventogenesis under submesophilic conditions, i.e., 18, 25, and 30°C using CO_2_ as carbon source and H_2_ as sole electron donor by the same anaerobic granular sludge as used by He et al. (2020). Besides, the effects of pH, carbon source (HCO_3_^–^ and glucose supplementation) and trace metals on ethanol production were also investigated.

## Materials and Methods

### Biomass

The anaerobic granular sludge was obtained from a 200 m^3^ UASB reactor producing methane from dairy industry effluent at 20°C and a hydraulic retention time (HRT) of 9–12 h. The total solid (TS) and volatile solid (VS) content was 42.7 (±1.0) g/L and 24.8 (±0.5) g/L, respectively. The granular sludge was first centrifuged at 5,500 rpm for 10 min to remove the supernatant and the pellet was heat- treated at 90°C for 15 min to select for spore forming acetogens as described by Dessì et al. (2017).

### Medium Composition

Medium was prepared according to Stams et al. (1993) and modified as follows: 408 mg/L KH_2_PO_4,_ 534 mg/L Na_2_HPO_4_⋅2H_2_O, 300 mg/L NH_4_Cl, 300 mg/L NaCl, 100 mg/L MgCl_2_⋅6H_2_O, 110 mg/L CaCl_2_⋅2H_2_O; 1 mL trace metal and 1 mL vitamin stock solution (Stams et al., 1993). 1 L medium (except for CaCl_2_⋅2H_2_O and vitamins) was prepared and brought to boiling in order to remove O_2_, cooled down to room temperature under an oxygen-free N_2_ flow, then CaCl_2_⋅2H_2_O and the vitamins were added as well as Na_2_S (0.24 g/L) as reducing agent.

### Experimental Set-Up

Batch tests were conducted in 125 mL serum bottles with 50 mL medium (gas: liquid ratio of 3:2) and granular sludge with an initial VS. concentration of 1.0 g/L. The bottles were sealed with rubber inlets and capped with aluminum crimp caps. A H_2_/CO_2_ (v/v, 80/20) gas mixture was injected by a gas exchanger system (GW-6400-3111, Germany) to an initial pressure of 1.8 (±0.15) bar (P_*H2*_ = 1.44 bar, P_*CO2*_ = 0.36 bar), in which 124.4 mL of the gas mixture was compressed in the 75 mL headspace. Blank experiments were set up with a H_2_/CO_2_ (v/v, 80/20) headspace without the granular sludge as well as a N_2_ (100%) headspace with the granular sludge (initial VS. concentration 1.0 g/L). At the start of the experiments, the gas pressure was measured every 24 h. H_2_/CO_2_ was injected when the gas pressure was detected below 1 bar. All experiments were performed in triplicates.

### Experimental Design

In order to enhance the C/H ratio of the substrate and thus enhance the electron donor utilization efficiency considering the substrate C/H ratio is 1/4 lower than the theoretical utilization ratio (Eq. 1), 2.1 g/L NaHCO_3_ (1.25 mmol carbon) was added in 50 mL medium to increase the CO_2_/H_2_ ratio to theoretically obtain a H_2_/CO_2_ ratio of 64/36 (v/v). 1 mL 1M HCl was also added to balance the pH increase by NaHCO_3_ (He et al., 2020).

The first batch test was set up at different temperatures (18, 25, and 30°C) using H_2_/CO_2_ or H_2_/CO_2_ with HCO_3_^–^. The second batch test was performed at 25°C at different pH 7, 6, and 5 and 0.3 g/L glucose with initial pH of 6. 0.3 g/L glucose + H_2_/CO_2_, H_2_/CO_2_ with no glucose and 0.3 g/L glucose with no H_2_/CO_2_ were supplied again when they were totally consumed. The third batch test was set up with different trace metal concentrations, namely 2 μM W (20×), 2 μM Mo (20×), 10 μM W (100×), 10 μM Mo (100×), 10 μM Ni (100×), and 50 μM Zn (100×) compared with 0.1 μM W, 0.1 μM Mo, 0.1 μM Ni and 0.5 μM Zn in the control. Incubations with medium with no trace metals were set up as control experiments.

Headspace (1 mL) and liquid (1 mL) samples were withdrawn from each vial every 24 h to analyze the gas and liquid phase. Liquid samples were centrifuged at 8,000 × g for 5 min and the supernatant was filtered with a syringe using a 0.22 μm PTFE-filter prior to analyzing ethanol and acetic acid concentrations.

### Analytical Methods

#### Gas-Phase Analysis

H_2_, CO_2_, and CH_4_ concentrations were measured using a HP 6890 gas chromatograph (GC, Agilent Technologies, United States) equipped with a thermal conductivity detector (TCD). The GC was fitted with a 15 m HP-PLOT Molecular Sieve 5A column (ID 0.53 mm, film thickness 50 μm). The oven temperature was kept constant at 60°C. The temperature of the injection port and the detector were maintained constant at 250°C. Helium was used as the carrier gas. Standard gas mixtures of CH_4_, H_2_, and CO_2_ were measured every time along with the sample measurements.

#### VFAs and Alcohols Analysis

VFA and alcohol concentrations were analyzed for each bottle from the liquid samples (1 mL) using high performance liquid chromatography (Agilent Co., United States) equipped with a refractive index detector (RID) and an Agilent Hi-Plex H column (Internal diameter × length, 7.7 × 300 mm, size 8 μm). A H_2_SO_4_ solution (5 mM) was used as mobile phase at a flow rate of 0.7 ml/min and with a sample injection volume of 50 μl. The column temperature was set at 60°C and the RID detector at 55°C. Total solid (TS) and volatile solid (VS) were measured according to standard methods (EPA, 2001). Calibration curves from 0.5 to 100 mM acetic acid, ethanol and butyric acid were made. The carbon (C) and electron (e^–^) recoveries were calculated according to our previous study (He et al., 2020).

## Results

### Effect of Initial pH on H_2_/CO_2_ Bioconversion at 25°C

**FIGURE 1:**
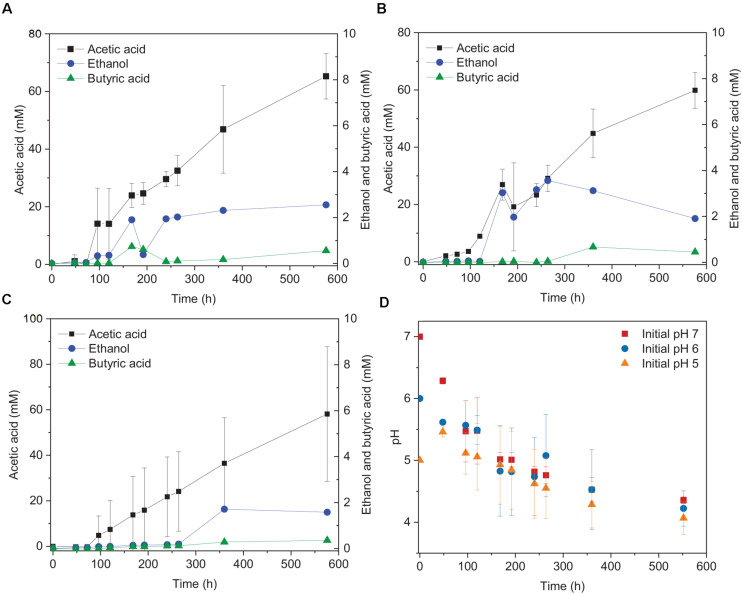
Acetic acid and ethanol production from H_2_/CO_2_ by heat-treated granular sludge at 25°C at **(A)** pH 7, **(B)** pH 6, **(C)** pH 5, and **(D)** change of pH over time.

The highest ethanol production was 2.5, 3.6, and 1.7 mM at initial pH of 7, 6, and 5, respectively (Table 1). The highest ethanol concentration was reached at an initial pH of 6, while the highest acetic acid concentration at pH 7 (Figures 1A,B and Table 1). A neutral initial pH enhanced the acetic acid production, but this may not be the best condition for ethanol production (Figure 1A). The pH decreased along with time: after 168 h of fermentation all pH values had decreased below 5 (Figure 1D). Ethanol production was detected at 120 h for the batches with initial pH of 7 and 6, while for pH 5, ethanol was observed at 360 h (Figure 1). It was noted that at initial pH 5, the acetic acid concentration reached 7.4 mM at 120 h, similar for the batch experiment at pH 6 with an acetic acid concentration of 9.0 mM, but ethanol production was not observed (Figures 1B,C). The C and e- recovery were, respectively, 123.6 and 123.1% at pH 5, 151.8, and 137.1% at pH 6, 112.6, and 116.0% at pH 7 (Table 1). A small amount of acetic acid (data not shown) production was detected in the control bottles without H_2_/CO_2_ (with 100% N_2_) (Table 2).

**TABLE 1:** Effect of pH, carbon supplements and trace metals on the maximum acetic acid and ethanol concentrations and H_2_ and CO_2_ consumption from H_2_/CO_2_ by heat-treated anaerobic granular sludge (at the end of incubation).

Conditions/Substrate	Products (mM)									
	
	Acetic acid	Butyric acid	Ethanol	CO_2_	H_2_	CH_4_	H_2_ consumption (%)	CO_2_ consumption (%)	C recovery (%)	e^–^ recovery (%)
pH 7	65.3 ± 7.9	0.6	2.6 ± 3.1	−46.9^a^	−96.7^a^	0	35.5	69.0	123.6	123.1
pH 6	59.9 ± 6.3	0.7	8.5 ± 3.1	−38.0^a^	−86.1^a^	0	31.5	55.7	151.8	137.1
pH 5	58.1 ± 29.6	0.4	1.7 ± 2.7	−40.3^a^	−81.7^a^	0	29.4	58.0	112.6	116.0
Glucose	14.8 ± 0.3	3.3 ± 0.2	0.6 ± 0.5	−^b^	−^b^	0	−^b^	−^b^	89.1	99.6
Glucose+H_2_/CO_2_	44.9 ± 11.5	3.7 ± 0.5	5.7 ± 2.4	−39.3^a^	−104.9^a^	0	37.5	56.2	89.0	80.4
Control	38.4 ± 15.8	0	7.6 ± 4.3	−42.7^a^	−80.8^a^	0	26.8	56.5	141.2 ± 26.2	176.5 ± 20.2
2 μM W	33.3 ± 13.8	0	3.9 ± 2.6	−36.9^a^	−81.5^a^	0	26.6	48.2	130.0 ± 18.7	145.6 ± 52.8
10 μM W	53.0 ± 4.4	0	14.8 ± 10.2	−48.5^a^	−107.1^a^	0	34.9	63.3	153.0 ± 20.0	172.7 ± 22.8
2 μM Mo	40.2 ± 16.0	0	11.3 ± 2.1	−47.2^a^	−91.7^a^	0	31.5	64.6	141.9 ± 25.1	178.4 ± 32.8
10 μM Mo	66.9 ± 11.0	0	1.4 ± 1.1	−51.0^a^	−95.5^a^	0	31.2	66.7	174.9 ± 15.8	204.1 ± 9.8
10 μM Ni	42.7 ± 6.9	0	3.7 ± 2.2	−40.3^a^	−70.9^a^	0	23.2	52.8	150.5 ± 13.5	197.1 ± 20.9
50 μM Zn	28.5 ± 7.7	0	3.3 ± 2.3	−34.0^a^	−47.9^a^	0	16.6	47.4	122.6 ± 12.0	203.5 ± 30.0

*^*a*^Negative values indicate an overall consumption of component during the experiment.*

*^*b*^Data not known since carbon is excess.*

**TABLE 2:** Effect of HCO_3_^–^ on the maximum acetic acid and ethanol concentration from H_2_/CO_2_ by heat-treated anaerobic granular sludge at initial pH 6.

System conditions	Substrate					
	
	H_2_/CO_2_			H_2_/CO_2_+HCO_3_^–^		
		
	18°C	25°C	30°C	18°C	25°C	30°C
**Products (mM)**						
Acetic acid	6.5 ± 2.6	29.7 ± 3.3	27.0 ± 2.4	24.3 ± 31.2	122.7 ± 5.8	81.3 ± 23.3
Ethanol	0.1	8.7 ± 9.2	3.6 ± 3.7	0.1	6.3 ± 7.8	4.3 ± 7.0
CO_2_	29.3	35.8	38.5	−	−	−
H_2_	73.3	115.6	95.0	−	−	−
Undissociated acid	−	22 ± 0.1	19 ± 3.2	−	36 ± 7.5	16 ± 12.5
H_2_ consumption (%)	26.4	41.7	34.0	−	−	−
CO_2_ consumption (%)	42.3	51.6	55.3	−	–	−
C recovery (%) ^*a*^	25.5 ± 10.2	120.4 ± 36.9	88.5 ± 20.0	−	−	−
e^–^ recovery (%) ^*b*^	20.5 ± 8.2	82.3 ± 31.0	75.5 ± 20.0	−	−	−
**Highest rate (mmoL⋅L^–1^⋅h^–1^)**						
H_2_ consumption	0.455	1.160	0.492	−	−	−
CO_2_ consumption	0.188	0.411	0.229	−	−	−
CH_4_	0	0	0	0	0	0
Acetic acid	0.145	0.32	0.289	0.97	0.79	1.58
Ethanol	0.00	0.11	0.05	0.00	0.09	0.10

**^*a*^Carbon recovery. ^*b*^Electron recovery.**

### Effect of Temperature on H_2_/CO_2_ Fermentation by Granular Sludge

**FIGURE 2:**
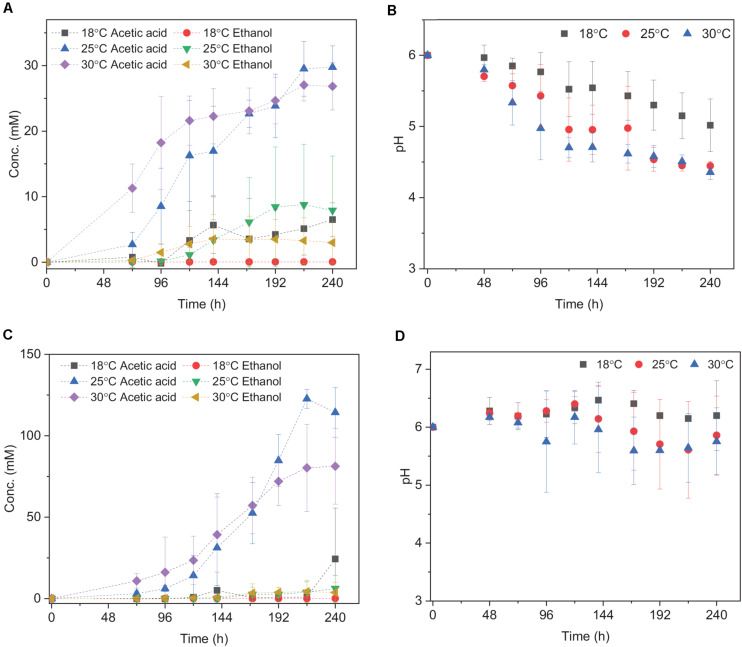
Acetic acid and ethanol yield and pH change using **(A,B)** H_2_/CO_2_; **(C,D)** H_2_/CO_2_ + HCO_3_^–^ by heat-treated granular sludge at 18, 25, and 30°C. Every point shown in the graphs is calculated as the average of three independent batch cultures, error bars indicate the standard deviation of the triplicates.

Acetic acid was the main fermentation product with the highest acetic acid concentration of 6.5 (±2.6), 29.7 (±3.3), and 27.0 (±2.4) mM at 18, 25, and 30°C, respectively (Table 2). The pH decreased along with the acetic acid accumulation from initially pH 6 to 5.0, 4.4, and 4.4 at 18, 25, and 30°C (Figure 2B), respectively. The highest ethanol concentration of 8.8 mM was obtained at 25°C with the highest average production rate of 0.03 mmol L^–1^ h^–1^ (Table 2). Ethanol started to be produced when acetic acid was more than 16.3 and 21.6 mM and the pH was lower than 4.9 at 25°C and 4.7 at 30°C, respectively (Figure 2A). The highest ethanol production rate was 0.11 mmoL⋅L^–1^⋅h^–1^ after 140 h of incubation at 30°C, while the highest acetic acid production rate was 0.32 mmoL⋅L^–1^⋅h^–1^ after 120 h of incubation at 25°C (Supplementary Figure 1 and Table 2).

The mesophilic (30°C) or psychrophilic (18°C) temperatures with an initial incubation pH of 6.0 negatively affected the ethanol production. The highest C and e^–^ recovery obtained at 25°C were 120.4 (±36.9)% and 82.3 (±31.0)%, respectively (Table 2). The acetic acid concentration after 72 h of incubation varied at 0.8, 2.7, and 11.2 mM in the18, 25, and 30°C incubations, respectively (Figure 2A), which demonstrated that higher temperatures reduced the lag phase of the acetic acid production. The average acetic acid and ethanol production rate were much lower at 18°C than at 25 or 30°C (Table 2). At 30°C, the C and e^–^ recovery were 88.5 (±20.0)% and 75.5 (±20.0)%, respectively, which were both lower than at 25°C. The lowest C and e^–^ recovery of 25.5 (±10.2)% and 20.5 (±8.2)%, respectively, were observed at 18°C (Table 2).

Upon the addition of HCO_3_^–^, the acetic acid production was highly enhanced at 25 and 30°C, while the ethanol production was below 4 mM (Figure 2C). Up to 122.7 (±5.8) mM acetic acid was obtained, which was 4.1-fold more than the highest acetic acid concentration (29.7 ± 3.3 mM) without HCO_3_^–^ addition at 25°C (Table 2). Similarly, the highest acetic acid concentration with HCO_3_^–^ was 3.0-fold higher than without HCO_3_^–^ at 30°C (Table 2). The highest acetic acid production rate amounted to 0.97, 0.79, and 1.58 mmoL⋅L^–1^⋅h^–1^ at 18, 25, and 30°C, respectively, which are all higher than in the absence of HCO_3_^–^ (Supplementary Figure 1B and Table 2). The pH increased initially from 6 to 6.2 at 120 h at 18, 25, and 30°C. At the end of the incubation, the pH varied between 6.2 and 6.5 at 18°C, decreased to 5.6 at 25°C and 5.3 at 30°C (Figure 2D). Overall, the pH kept stable between 5.2 and 6, although the acetic acid concentration was much higher than in the absence of HCO_3_^–^ (Figure 2D).

### Effect of Glucose on H_2_/CO_2_ Bioconversion at 25°C

**FIGURE 3:**
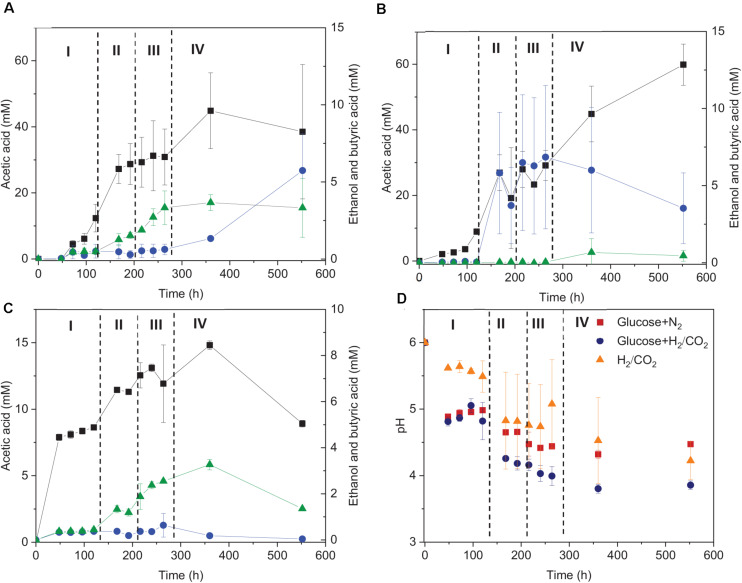
Acetic acid, ethanol and butyric acid production by heated-treated granular sludge at 25°C using **(A)** glucose+H_2_/CO_2_, **(B)** H_2_/CO_2,_ and **(C)** glucose as the substrate and **(D)** change of pH. The dashed vertical lines represent the different phases in the fermentation process; I: 0–120 h, II: 120–192 h, III: 192–264 h, IV: 264–552 h.

The fermentation process using solely H_2_/CO_2_ could be separated in four stages (Figure 3): stage I (0–120 h) is the acetic acid accumulation phase, stage II (120–192 h), and III (192–264 h) represent, respectively, the quick acetic acid production and butyric acid accumulation, whereas ethanol was produced in stage IV (264–552 h). In stage I, when using glucose + H_2_/CO_2_ as the substrate, acetic acid was not detected after 48 h of incubation, then 16.5 mM acetic acid and 0.45 mM butyric acid were observed at 120 h. Thereafter, acetic acid production rate reached to its maximum (0.32 mmoL⋅L^–1^⋅h^–1^) at 168 h in stage II (Figure 3A). The acetic acid concentration kept relatively stable during stage III, during which the butyric acid concentration started to increase (from 0.5 to 3.3 mM) (Figure 3A). Ethanol started to increase during stage IV and reached to 5.7 (±2.4) mM when the pH decreased below 4 (Figures 3A,D).

When using glucose as the sole substrate, the acetic acid concentration reached 7.9 mM at 48 h. The highest acetic acid concentration reached 14.8 mM after 360 h of incubation. The highest butyric acid (3.3 mM) and ethanol (0.6 mM) concentrations were obtained at 360 h (Figure 1C and Table 1). The distinct change when adding glucose was the quick decrease in pH from initially 6 to 4.8 after 48 h of incubation (Figure 3D). During stage II and III, the pH decreased quickly to below 4 for the batches with both glucose and H_2_/CO_2_ and to 4.5 for the glucose only batches (Figure 3D). Compared to the H_2_/CO_2_ solely incubation (Figure 3B), the addition of glucose enhanced the butyric acid production. The C and e^–^ recovery were, respectively, 89.1 and 99.6% in the batches with glucose, compared to 89.0 and 80.4% for glucose + H_2_/CO_2_ (Table 1).

### Effect of Trace Metals (W, Mo, Zn, Ni) on H_2_/CO_2_ Fermentation at 25°C

**FIGURE 4:**
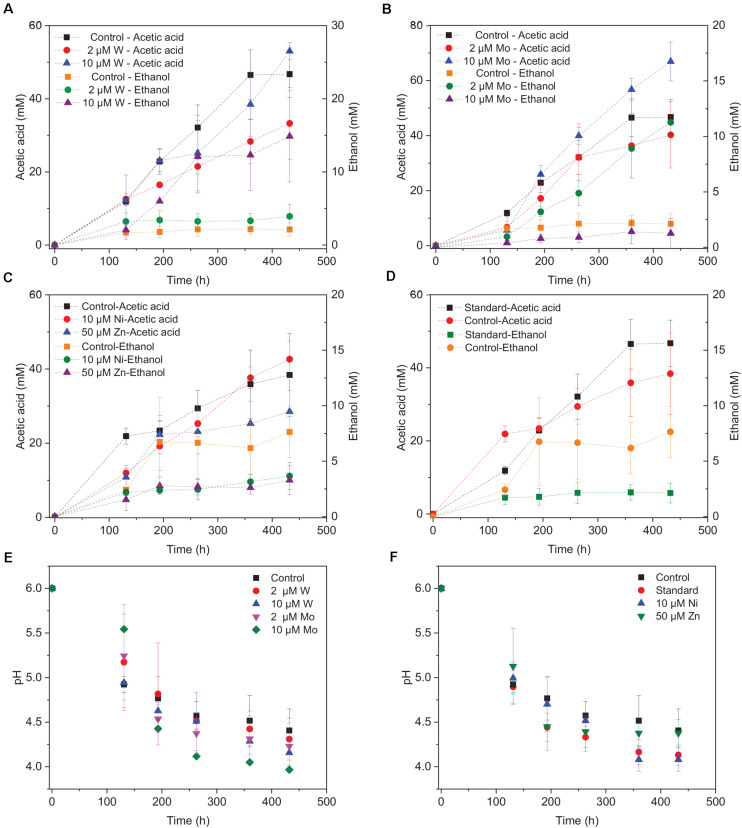
Acetic acid and ethanol production by heat-treated granular sludge using H_2_/CO_2_ as the substrate at 25°C with the addition of **(A)** 2 μM, 10 μM W, **(B)** 2 μM, 10 μM Mo, **(C)** 10 μM Ni, 50 μM Zn, **(D)** No trace metals, and **(E,F)** change of pH.

**FIGURE 5:**
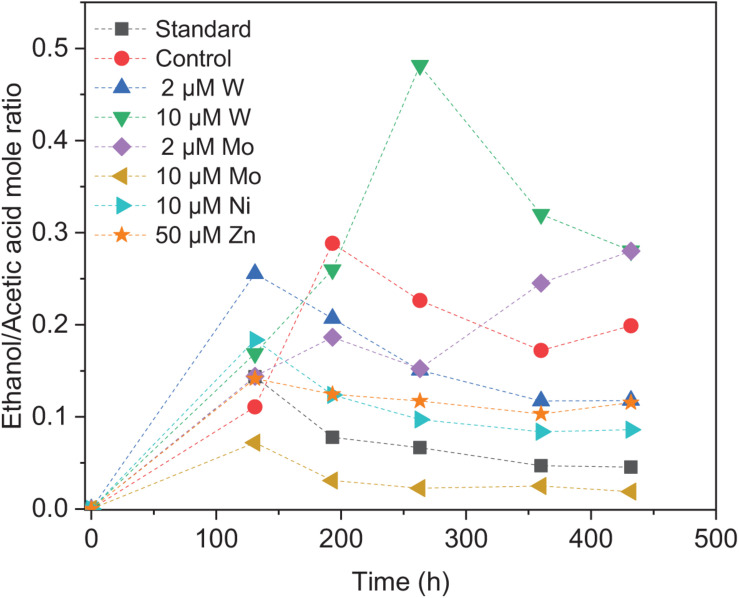
Ethanol to acetic acid mole ratio by heat-treated granular sludge using H_2_/CO_2_ as the substrate at 25°C with the standard medium, control and addition of 2 μM, 10 μM W, 2 μM, 10 μM Mo, 10 μM Ni, and 50 μM Zn.

**FIGURE 6:**
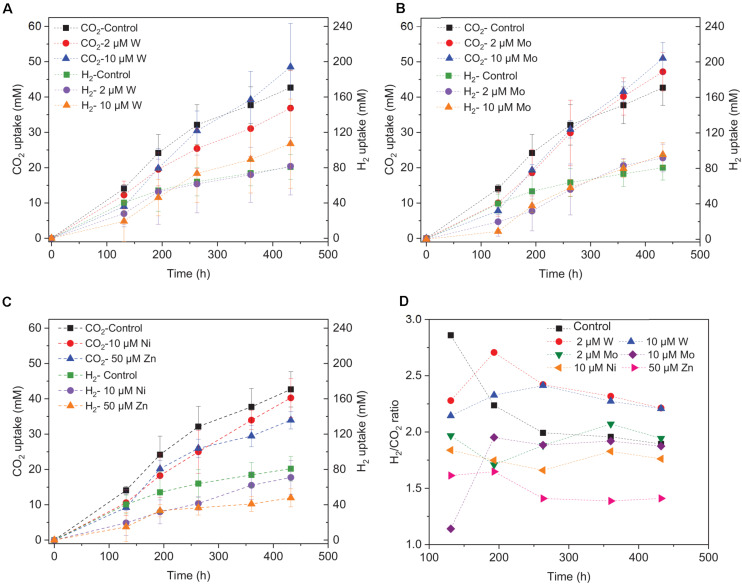
H_2_ and CO_2_ uptake with the addition of **(A)** 2 μM, 10 μM Mo, **(B)** 2 μM, 10 μM W, **(C)** 10 μM Ni, 50 μM Zn, and **(D)** H_2_/CO_2_ uptake ratio by heat-treated granular sludge using H_2_/CO_2_ as the substrate at 25°C with initial pH 6.

Upon the addition of 2 and 10 μM W, acetic acid production constantly increased to the highest concentration of 33.3 (±13.8) and 53.0 (±4.4) mM, respectively (Figure 4A). Ethanol kept increasing after 120 h with the addition of 10 μM W and reached 14.8 (±10.2) mM (Figure 4A). The addition of 10 μM W also reached the highest mole ratio of ethanol to acetic acid of 0.48 at 263 h and 0.28 at the end of the incubation (Figure 5). With the addition of 2 μM W, ethanol increased to 3.2 mM then kept relatively stable till 3.9 (±2.6) mM at the end of the incubation (Figure 4A). The addition of 2 μM W reached the highest mole ratio of ethanol to acetic acid of 0.26 at 131 h and 0.12 at the end of the incubation (Figure 5). The addition of 10 μM W enhanced ethanol production up to 3.8 and 7.0-fold than with, respectively, 2 μM W and the control. Upon the addition of 2 and 10 μM W, H_2_ consumption was, respectively, 81.5 (±32.5) and 107.1 (±50.5) mM (Figure 6A), which are both higher than in the absence of trace metals (80.8 ± 14.0 mM, Figure 6A). The addition of 10 μM W to the medium enhanced the ethanol production with the highest ethanol to acetic acid ratio of 0.48.

Upon addition of 2 and 10 μM Mo, the acetic acid concentration constantly increased to the highest value of 40.2 (±16.0) and 66.9 (±11.4) mM, respectively (Figure 4B). Ethanol kept increasing with the addition of 2 μM Mo and reached 11.3 (±2.1) mM at the end of the incubation (Figure 4B). However, ethanol was not significantly produced, with the maximum concentration of 1.4 (±1.1) mM with 10 μM Mo. The addition of 2 μM Mo enhanced ethanol production up to 8.1 and 5.4-fold, respectively, compared to 10 μM Mo and the control. The acetic acid production with the addition of 2 and 10 μM Mo was, respectively, 6.7 and 5.6 mM at 120 h, which was lower than the 11.7 mM produced by the control (Figure 4B). The acetic acid concentration increased quickly to a higher acetic acid concentration with 10 μM Mo compared to with 2 μM Mo and the control after 120 h. The pH decreased along with the accumulation of acetic acid (Figure 4E). The addition of 2 μM Mo reached the highest ethanol to acetic acid ratio of 0.28, while the ratio was 0.02 for 10 μM Mo addition at the end of the incubation (Figure 5).

With the addition of 10 μM Ni, the highest acetic acid and ethanol concentration amounted to, respectively, 42.7 (±6.9) and 3.7 (±2.2) mM (Figure 4C). The highest acetic acid and ethanol concentration with the addition of 50 μM Zn were 28.5 (±7.7) and 3.3 (±2.3) mM, respectively. Either 10 μM Ni or 50 μM Zn did not significantly enhance the ethanol production. The presence of 50 μM Zn inhibited the acetic acid production compared to the control (Figure 4C).

Surprisingly, the ethanol concentration in the incubation without trace metal supplementation is higher than with the control (Figure 4D). The highest acetic acid and ethanol concentration reached to 46.7 (±8.3) and 2.1 (± 1.9) mM, respectively, in the control. With no trace metals addition, ethanol started to be produced after 131 h and quickly increased to 6.7 mM, and then slightly increased to 7.6 mM at the end of the incubation. Ethanol production started at 131 h with a concentration of 1.7 mM, and then kept stable till the end of the incubation (2.1 mM) in the control (Figure 4D).

Overall, the acetic acid production was enhanced by the addition of 10 μM Mo, followed by 10 μM W and 2 μM Mo, whereas the presence of 50 μM Zn, 2 μM W, 10 μM Ni or the absence of trace metals inhibited acetic acid production compared to the control (Figure 4). The ethanol production was the highest in the presence of 10 μM W, followed by 2 μM Mo, while the absence of trace metals reached a higher ethanol production than the 10 μM Ni, 10 μM Mo, 2 μM W, 50 μM Zn, and control incubation (Figure 4).

The decrease in pH corresponded to the accumulation of acetic acid. In the presence of 10 μM Mo, the pH reached the lowest value at the end of the incubation and the acetic acid concentration reached the highest compared to 2 μM and the control (Figure 4E). Ethanol production started after 120 h; the pH varied from 4.75 to 5.5 (Figure 4E). In the presence of 2 μM Mo and 10 μM W, ethanol production was enhanced even though the pH dropped to 4.2 (Figure 4E). The cumulative H_2_ uptake in all media was between 211.7 and 245.3 mM. The cumulative CO_2_ uptake with different trace metal concentrations was between 61.4 and 68.7 mM (Figure 6).

## Discussion

### Effect of Temperature and pH on Solventogenesis

This study showed that the highest ethanol concentration was produced at an initial pH of 6 at 25°C in the H_2_/CO_2_ incubations. An initial pH of 6 favored the ethanol production compared to pH 7 and 5 from H_2_/CO_2_ by granular sludge (Figure 1). Ethanol production via solventogenesis is linked to the accumulation of undissociated acetic acid and pH (Richter et al., 2016). Solventogenesis occurs when the pH decreases to 4.5–5 and the undissociated acetic acid is able to cross the cytoplasmic membrane by diffusion: alcohol formation then avoids cell damage or death by the protons that would be released by dissociated acetic and butyric acids (Baronofsky et al., 1985; Jones and Woods, 1986; Richter et al., 2016). It should be noted that a low pH can stimulate ethanol production, however, incubations conducted with initial pH 5 did not reach higher ethanol concentrations than the incubation with initial pH 6. This could be because pH 6 facilitated cell growth and reached higher acetic acid concentrations than at pH 5 (Figure 1). Considering the acetic acid concentration of pH 5 and 6 were lower than the pH value that induces an “acid crash” (Mohammadi et al., 2011), the higher acetic acid concentration at pH 6 may obtain a higher ethanol production than at pH 5. Besides, fermentation at an initial pH 5 may have provided an unfavorable environment for cell growth, since the growth pH for the known autotrophic *Clostridium* sp. ranges from pH 5 to 7 (Fernández-Naveira et al., 2017a). Kundiyana et al. (2011) studied the ethanol production by *C. ragsdalei* from 10 g corn steep liquor purged daily with syngas (5% H_2_, 15% CO_2_, 20% CO) with initial incubation at pH 7, 6, and 5 at 32, 37, and 42°C. Without a buffer, their experiment at initial pH 5 produced less ethanol than at pH 7 and 6 at 32°C (Kundiyana et al., 2011).

With an initial pH of 6, submesophilic temperatures (25°C) enhanced ethanol production from H_2_/CO_2_ by granular sludge in this study (Figure 2). The growth temperature of most acetogens ranges from 20 to 42°C, with the optimum at 37°C (Naik et al., 2010; Munasinghe and Khanal, 2010). Fermentation at 25°C, which is below the optimum temperature, might slow down microbial metabolism and hence avoid the “acid crash” phenomenon. Solventogenesis is negatively affected or even terminated by a sharp increase of undissociated acids, a so called “acid crash” (Ramió-Pujol et al., 2015). Such an acid crash can be mitigated by slowing down the microbial metabolism, e.g., by lowing the temperature, thus reducing the acid accumulation rates. Similarly, 10 g corn steep liquor and syngas (5% H_2_, 15% CO_2_, 20% CO) were fermented by *C. ragsdalei* at 32, 37, and 42°C and 1.89 g/L of ethanol was obtained at 32°C, which is 2.7 fold higher than at 37°C (0.69 g/L) with an initial incubation pH of 6.0 (Kundiyana et al., 2011). The temperature of 18°C is lower than the reported growth temperatures for most of acetogens (Mohammadi et al., 2011), which likely caused the lower acetic acid production than at 25 and 37°C. Our previous tests demonstrated the *Clostridium* genus was successfully enriched under mesophilic and submesophilic conditions using the same inoculum (He et al., 2020). Chakraborty et al. (2020) demonstrated enhanced ethanol production from C_1_ gas by granular sludge and *Clostridium autoethanogenum* was successfully enriched at 33°C. Similarly, Samanides et al. (2020) reported an increased relative abundance of *Clostridium* of 65.9% in anaerobic granular sludge for acetic acid production, when first exposed to heat (95°C for 30 min) and incubated with 100% CO_2_ and 100 g/L zero valent iron at 33°C.

The higher C recovery in acetic acid and ethanol production from CO_2_ (Table 1) can be attributed to the fact that granular sludge used as inoculum contains a certain amount of calcium carbonate precipitates. Calcium carbonate can precipitate both in the core and on the surface of granular sludge and the surface part of the calcium carbonate precipitates contributes to the aggregation of the granules (Yang et al., 2010). UASB sludge can reach a calcium carbonate content of up to 90% of the ash content in anaerobic wastewater treatment systems (Van Langerak et al., 1998). The high C recovery is in accordance with our previous results using the same UASB sludge (He et al., 2020). The carbon released from the calcium carbonate precipitates results in a positive carbon balance.

Methane was not observed during the whole incubation (Supplementary Table 3), which was attributed to the heat-pretreatment and initial pH of 6. Similarly, Modestra et al. (2020) reported that both heat and acid treatment of granular sludge inhibited methane production and enriched for homoacetogenic bacteria when using gaseous substrate H_2_/CO_2_.

### Effect of Organic and Inorganic Carbon Source on Solventogenesis

Upon HCO_3_^–^ addition to increase the C/H ratio, acetic acid production by granular sludge from H_2_/CO_2_ was enhanced at 18, 25, and 30°C, but not ethanol production. The failure of enhanced ethanol production could be due to the higher pH caused by the HCO_3_^–^ buffering capacity. Ethanol production is triggered at low pH, for instance, 4.5–5 (Ganigué et al., 2016). However, the additional HCO_3_^–^ acts as a buffer and prevents the pH decreasing sharply. The high acetic acid and lower ethanol production might thus be attributed to the higher pH: 5.2 and 6 for, respectively, without and with HCO_3_^–^ addition than without HCO_3_^–^ addition (Figure 2D). On the other hand, HCO_3_^–^ offered extract carbon and increased the acetic acid production. Maddox et al. (2000) reported undissociated acid formation above 57–60 mM induced an “acid crash.” However, the highest undissociated acetic acid concentrations obtained in this study were 36 and 16 mM at 25 and 30°C, respectively (Table 2), thus lower than the reported value at which an acid crash occurs. Upon the addition of HCO_3_^–^, the *Clostridium* genus had a similar relative abundance compared to without HCO_3_^–^ addition at 25°C from the same inoculum (He et al., 2020).

Glucose enhances the growth of *C. autoethanogenum* and *C. carboxidivorans* (Fernández-Naveira et al., 2017a; Cheng et al., 2019). Addition of 0.3 g/L glucose enhanced the acetic and butyric acid production, but not the ethanol production by the granular sludge. 0.3 g/L glucose was added at the beginning of each of the four phases, from which 20 mM acetic acid or 13.3 mM butyric acid can be produced when the carbon from glucose is totally converted to acids. The inhibited ethanol production can be attributed to the different conversion pathway of the organic substrate glucose and the inorganic substrate H_2_/CO_2_. Indeed, the addition of glucose introduces another pathway: the glycolysis pathway. Marcellin et al. (2016) investigated the energy metabolism of a model acetogen *C. autoethanogenum* showing that during heterotrophic growth, cells relied mainly on the Embden–Meyerhof–Parnas (EMP) glycolysis pathway, whereas under autotrophic conditions exclusively the WLP pathway is used. The energy yield (ATP and redox state) is, however, unaffected between heterotrophic and autotrophic growth. Fernández et al. (2017) investigated the glucose (30 g/L) bioconversion profile at constant pH 6.2 and 5.2 by *Clostridium carboxidivorans*. Acetic acid was formed as the first metabolite and butyric acid appeared a few hours later and kept increasing, while ethanol was produced during the acidification stage at pH 6.2. Fernández et al. (2017) also found that the glucose consumption stopped after 72 h at pH 5.2. In this study, when the pH dropped to below 4 during stage IV (Figure 3D), increasing concentrations of acetic acid, butyric acid and ethanol were observed (Figure 3A). It should be noted that a small amount of ethanol was produced at a pH value as low as 4 by the granular sludge used in this study (Figure 3A), which is seldom reported before. Considering the mixed culture inoculum, one possible reason might be that heterotrophic acetogens consumed the glucose first and autotrophic acetogens adapted later and produced ethanol at the end of the incubation.

### Enhanced Ethanol Production by Trace Metal Addition

Ethanol production from H_2_/CO_2_ by granular sludge was enhanced by the addition of 2 μM Mo or 10 μM W, while the addition of 10 μM Mo, 2 μM W, 10 μM Ni, and 50 μM Zn did not significantly affect the ethanol production. There are only few studies on the effect of trace metals on ethanol production by mixed cultures compared to studies using pure cultures. Saxena and Tanner (2011) increased the SeO_4_^2–^ and WO_4_^2–^ concentration to 5.3 and 6.8 μM, respectively, which resulted in an increased ethanol production from synthesis gas by *Clostridium ragsdalei* from 35.73 mM under standard metal concentrations to 54.4 and 72.3 mM, respectively, upon SeO_4_^2–^ and WO_4_^2–^ addition. They also observed that ethanol production decreased to 23.64 mM at higher concentrations of Mo (8.3 μM). The highest mole ratio of ethanol to acetic acid of 0.48 with 10 μM W is in accordance with the highest ethanol production in this study (Figure 5). Abubackar et al. (2015) investigated the carbon monoxide fermentation by *Clostridium autoethanogenum* and obtained the highest ethanol to acetic acid ratio of 0.19 in experiments with 0.75 μM W.

Saxena and Tanner (2011) found that ethanol production from synthesis gas by *C. ragsdalei* decreased to 22.02 and 1.55 mM when Fe, Co, Mo, and W were eliminated from the medium. This study, however, showed that without trace metals, the ethanol production was enhanced compared to the control. Nutrient-stress conditions such as lack of trace elements may also stimulate the shift from acetic acid to ethanol. Richter et al. (2016) performed proteomic and metabolomic analyses on a two-stage syngas fermentation (*Clostridium ljungdahlii)* system and did not find a difference in the abundance of enzymes of the central metabolic pathways, concluding that nutrient limitation and the resulting growth limitation redirect reducing equivalents toward ethanol production. The H_2_ to CO_2_ consumption ratio varies between 2.1 and 2.5 (Figure 6D) and thus conform the production of a mixture of acetic acid and ethanol (Eqs. 1, 2, Table 1). The trace metals affected the enzymes such as FDH, AOR, and ADH to catalyze acetic acid and ethanol production and thus strengthen the homoacetogens, such as the *Clostridium* genus as reported by He et al. (2020). The W-containing AOR enzyme has been reported in *Clostridium thermoaceticum* (Strobl et al., 1992). Besides, W can serve as a potential acting element for CO_2_ reduction FDHs, W even becomes an essential element for nearly all enzymes of the AOR family (Fernández-Naveira et al., 2019). This study reported lower ethanol production with Mo than W, despite the close chemical similarities between Mo and W. However, it has been demonstrated that W, different than Mo, can be selectively transported into some prokaryotic cells by two ABC-type transporters that contain the binding protein TupA or WtpA (Andreesen and Makdessi, 2008).

## Conclusion

The optimum conditions for ethanol production by anaerobic granular sludge using H_2_/CO_2_ as the substrate were 25°C and an initial pH of 6. An initial pH of 7 enhanced acetic acid production, while an initial pH of 5 totally inhibited ethanol production. The use of glucose and CO_2_ as co-substrate enhanced butyric acid production (3.3 mM), while ethanol production occurred at a pH as low as 4. The presence of 10 μM W and 2 μM Mo enhanced the ethanol production by 7.0 and 5.4-fold, respectively.

## Data Availability Statement

The original contributions presented in the study are included in the article/Supplementary Material, further inquiries can be directed to the corresponding author/s.

## Author Contributions

YH carried out all experimental incubations, data analysis, and drafted the manuscript. CC conceived the study, participated in its design and coordination, and reviewed the manuscript. PL conducted the project supervision and the manuscript revision. All authors contributed to the article and approved the submitted version.

## Conflict of Interest

The authors declare that the research was conducted in the absence of any commercial or financial relationships that could be construed as a potential conflict of interest.
