# Supplementary material for: Bioethanol Production From H2/CO2 by Solventogenesis Using Anaerobic Granular Sludge: Effect of Process Parameters
Source: Front Microbiol. 2021 Mar 10;12:647370. doi: 10.3389/fmicb.2021.647370 (PMC8006448; doi:10.3389/fmicb.2021.647370)
Supplement: Supplementary file 1 [file Data_Sheet_1.docx]

Appendix

Bioethanol production from H_2_/CO_2_ by solventogenesis using anaerobic granular sludge: effect of process parameters

Yaxue He*, Chiara Cassarini, Piet N. L. Lens

| Gas | Time/h | pH 7 | | | | pH 6 | | | | pH 5 | | | |
| --- | --- | --- | --- | --- | --- | --- | --- | --- | --- | --- | --- | --- | --- |
|  |  | mmol | stdev | mM | stdev | mmol | stdev | mM | stdev | mmol | stdev | mM | stdev |
| H_2_ | 120 | 2.72 | 2.82E-05 | 38.86534 | 0.402144 | 3.32 | 3.44E-06 | 47.4042 | 0.049114 | 3.42 | 6.41E-05 | 48.81247 | 0.915895 |
|  | 192 | 3.14 | 0.000135 | 44.90011 | 1.921857 | 2.71 | 6.04E-05 | 38.67069 | 0.862869 | 3.63 | 3.25E-05 | 51.92606 | 0.464015 |
|  | 264 | 3.22 | 3.8E-06 | 46.02693 | 0.05429 | 3.67 | 3.54E-06 | 52.40907 | 0.050599 | 3.53 | 5.9E-06 | 50.4829 | 0.08428 |
|  | 360 | 3.20 | 4.68E-05 | 45.6503 | 0.6692 | 3.40 | 4.05E-05 | 48.55105 | 0.578993 | 3.16 | 1.96E-05 | 45.16212 | 0.280669 |
| CH_4_ | 120 | 0.00 | 0 | 0 | 0 | 0.00 | 0 | 0 | 0 | 0.00 | 0 | 0 | 0 |
|  | 192 | 0.00 | 9.54E-09 | 0.000154 | 0.000136 | 0.00 | 0 | 0 | 0 | 0.00 | 0 | 0 | 0 |
|  | 264 | 0.00 | 0 | 0 | 0 | 0.00 | 0 | 0 | 0 | 0.00 | 2.47E-09 | 0.000205 | 3.52E-05 |
|  | 360 | 0.00 | 0 | 0 | 0 | 0.00 | 3.61E-09 | 0.000111 | 5.15E-05 | 0.00 | 0 | 0 | 0 |
| CO_2_ | 120 | 0.09 | 2.82E-05 | 1.305274 | 0.402144 | 0.46 | 3.44E-06 | 6.501506 | 0.049114 | 0.31 | 6.41E-05 | 4.407774 | 0.915895 |
|  | 192 | 0.35 | 0.000135 | 5.071381 | 1.935333 | 0.35 | 6.04E-05 | 5.030698 | 0.862869 | 0.48 | 3.25E-05 | 6.85547 | 0.464015 |
|  | 264 | 0.59 | 3.8E-06 | 8.473701 | 0.05429 | 0.75 | 3.54E-06 | 10.73097 | 0.050599 | 0.73 | 5.87E-06 | 10.45095 | 0.083858 |
|  | 360 | 0.44 | 4.68E-05 | 6.250759 | 0.6692 | 0.56 | 4.04E-05 | 7.981951 | 0.577147 | 0.52 | 1.96E-05 | 7.489061 | 0.280669 |

**Table 1** H_2_, CO_2_ and CH_4_ concentration at initial pH 5, 6 and 7 by heated-treated granular sludge at 25°C using H_2_/CO_2_ as the substrate.

**Table 2** H_2_, CO_2_ and CH_4_ concentration at initial pH 5, 6 and 7 by heated-treated granular sludge using H_2_/CO_2_ as the substrate at 18, 25 and 30°C.

| Temperature | Time/h | H_2_ mmol average | H_2_ mM | Stdev | CO_2_ mmol average | CO_2_ mM | Stdev |
| --- | --- | --- | --- | --- | --- | --- | --- |
| 25°C | 96 | 2.92 | 41.75 | 0.00 | 0.69 | 9.88 | 0.00 |
|  | 140 | 2.27 | 32.38 | 0.00 | 0.60 | 8.55 | 0.00 |
|  | 170 | 2.52 | 35.94 | 14.98 | 0.37 | 5.35 | 4.20 |
|  | 240 | 3.60 | 51.45 | 4.95 | 0.68 | 9.74 | 1.73 |
| 30°C | 96 | 1.98 | 28.30 | 0.00 | 0.09 | 1.36 | 0.00 |
|  | 140 | 3.35 | 47.79 | 0.00 | 0.50 | 7.10 | 0.00 |
|  | 170 | 3.91 | 55.81 | 1.14 | 0.80 | 11.38 | 0.26 |
|  | 240 | 3.64 | 52.01 | 7.72 | 0.79 | 11.32 | 1.94 |
| 18°C | 96 | 3.12 | 44.54 | 0.00 | 0.45 | 6.48 | 0.00 |
|  | 140 | 3.66 | 52.28 | 0.00 | 0.69 | 9.85 | 0.00 |
|  | 170 | 4.00 | 57.09 | 2.77 | 0.84 | 12.05 | 1.03 |
|  | 240 | 3.50 | 49.94 | 2.10 | 0.81 | 11.61 | 0.75 |

**Table 3** H_2_, CO_2_ and CH_4_ concentration by heated-treated granular sludge using glucose and glucose+H_2_/CO_2_ as the substrate at 25°C.

| Gas | Time (h) | Glucose + N_2_ | | | | Glucose + H_2_ /CO_2_ | | | |
| --- | --- | --- | --- | --- | --- | --- | --- | --- | --- |
|  |  | mmol | stdev | mM | stdev | mmol | stdev | mM | stdev |
| H_2_ | 120 | 0.11 | 1.74E-06 | 1.556703 | 0.024828 | 3.10 | 7.91E-06 | 44.30131 | 0.112987 |
|  | 192 | 1.12 | 5.49E-05 | 15.98327 | 0.784498 | 3.17 | 2.32E-06 | 45.23741 | 0.033157 |
|  | 264 | 1.86 | 3.65E-07 | 26.50727 | 0.005215 | 3.21 | 2.06E-05 | 45.81995 | 0.294558 |
|  | 360 | 0.68 | 5.19E-07 | 9.771044 | 0.007416 | 2.75 | 3.32E-05 | 39.32282 | 0.474463 |
| CH_4_ | 120 | 0.00 | 0 | 0 | 0 | 0.00 | 0 | 0 | 0 |
|  | 192 | 0.00 | 0 | 0 | 0 | 0.00 | 0 | 0 | 0 |
|  | 264 | 0.00 | 4.26E-09 | 0.009858 | 6.09E-05 | 0.00 | 1.12E-08 | 0.000443 | 0.00016 |
|  | 360 | 0.00 | 0 | 0 | 0 | 0.00 | 0 | 0 | 0 |
| CO_2_ | 120 | 4.35 | 1.74E-06 | 62.19119 | 0.024828 | 0.49 | 7.91E-06 | 7.043617 | 0.112987 |
|  | 192 | 3.74 | 5.49E-05 | 53.40352 | 0.784498 | 0.52 | 2.32E-06 | 7.426709 | 0.033157 |
|  | 264 | 3.07 | 6.46E-07 | 43.85955 | 0.009225 | 0.65 | 1.99E-05 | 9.257158 | 0.284829 |
|  | 360 | 4.66 | 5.19E-07 | 66.58675 | 0.007416 | 0.48 | 3.32E-05 | 6.913478 | 0.474463 |
